# A Rare and Surprising Case of Spontaneous Type B Carotid-Cavernous Fistula in an Internal Medicine Ward

**DOI:** 10.7759/cureus.31456

**Published:** 2022-11-13

**Authors:** Ana Luísa Campos, Filipa Madalena F Gonçalves, Rosa Cardoso, Filipa Sampaio, Jorge Cotter

**Affiliations:** 1 Internal Medicine, Hospital da Senhora da Oliveira, Guimarães, PRT; 2 Ophthalmology, Hospital da Senhora da Oliveira, Guimarães, PRT

**Keywords:** angiography, superior ophthalmic vein, carotid artery, cavernous sinus, post-septal cellulitis, carotid-cavernous fistula

## Abstract

Carotid-cavernous fistulas are abnormal communications between the carotid arteries and the cavernous sinus. They can be spontaneous, which is rare, or acquired, most often post-traumatic. We describe the case of a 59-year-old woman with complaints of right-sided red eye and blurred vision that did not improve with antibiotic treatment for bacterial conjunctivitis, progressing to what appeared to be post-septal cellulitis. The patient had exuberant chemosis, diplopia, VI cranial nerve palsy, and elevated intraocular pressure in the right eye. Computed tomography of the orbits showed right-sided thickening of the soft tissues of the upper eyelid and the medial and lateral rectus muscles, and an enlargement of the ipsilateral superior ophthalmic vein. However, antibiotics did not cause any kind of improvement. After a laborious diagnostic march, the diagnosis of a rare case of Barrow type B spontaneous carotid-cavernous fistula was confirmed. The patient underwent confirmatory angiography with endovascular treatment at the same time, showing rapid improvement after the procedure, without any sequelae. It is of great importance that clinicians are alert to this diagnosis, as diagnostic and therapeutic delay can lead to severe ocular compromise. In patients with a presumptive diagnosis of conjunctivitis and/or orbital cellulitis that does not improve with antibiotic treatment, the differential diagnosis with this rare entity should be considered, so that the appropriate treatment can be timely instituted.

## Introduction

Carotid-cavernous fistulas (CCFs) are abnormal communications between the internal and/or external carotid arteries and the cavernous sinus (CS) leading to blood flow from the high-pressure arterial system to the low-pressure venous system of the CS. In direct CCFs, there is communication between the internal carotid artery (ICA) and the CS. In indirect CCFs, a rarer condition, the abnormal communication is between meningeal branches of the ICA and/or the external carotid artery (ECA) and the CS [1]. The most common cause of direct CCFs is head trauma. Spontaneous CCFs are uncommon [2].

The mixing of venous and arterial blood generates venous hypertension that is transmitted to the orbital contents, which manifests as proptosis, conjunctival hyperemia, episcleral venous engorgement (arterialization of episcleral veins with corkscrew-like blood vessels), chemosis, cranial nerve palsy with impaired eye movement, increased intraocular pressure (IOP), and sometimes, retinal hemorrhages [3]. Among the differential diagnoses of CCFs, we find post-septal (orbital) cellulitis [4]. The aim of this article was to describe a rare case of CCF resulting from abnormal communication between the meningeal branches of the ICA and the CS, whose presentation mimicked orbital cellulitis, representing a major diagnostic challenge.

## Case presentation

A 59-year-old woman went to the emergency department (ED), referred by her attending physician, with complaints of right frontal headache with one week of evolution and high blood pressure (BP). She had essential arterial hypertension, usually controlled with ramipril 5 mg, type 2 diabetes mellitus controlled with oral antidiabetics, depression, hypothyroidism, and diagnosis of breast cancer five years ago, treated with surgery and hormonotherapy, without signs of recurrence.

In the ED, blood pressure was 183/85 mmHg. Physical examination was normal. Blood tests, head computed tomography (CT), and electrocardiogram did not reveal any abnormality. After administration of analgesics for the headache, the patient was relieved from the pain, her BP decreased, and she was discharged with the recommendation of adjustment of antihypertensive medication by her attending physician. After 10 days the patient still had a headache. Edema and conjunctival hyperemia appeared in the right eye, as well as blurred vision, so the patient went to the ophthalmology ED. There was no history of head or eye trauma. Due to the suspicion of bacterial conjunctivitis, she was discharged with oral amoxicillin+clavulanic acid and ofloxacin+dexamethasone eye drops.

Five days later, there was no symptomatic improvement and the patient started with diplopia, so she went to the ophthalmologist again. Ophthalmological examination showed exuberant conjunctival chemosis and hyperemia, and limitation in adduction and abduction of the right eye. Fundoscopy showed some vascular tortuosity in the four right quadrants, but the optic disc was pink (no pallor) and well delimited bilaterally. IOP in the right eye was elevated (26 mmHg). No Tyndall effect was observed. There was no papillary or follicular reaction in the tarsal conjunctiva, foreign bodies, or exudate. The pupils were isochoric, and direct and consensual pupillary reflexes were present. Visual fields were also preserved. Visual acuity in the right eye was 20/40.

**Figure 1:**
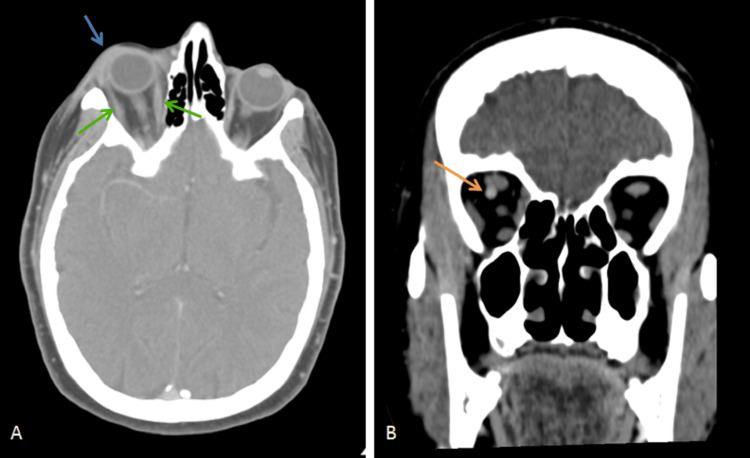
Orbital computed tomography images of the patient. (A) Axial view showing the inflammation of the upper right eyelid soft tissues and right eyeball proptosis (blue arrow), and the swelling of the medial and lateral rectus muscles (green arrows) and (B) coronal view showing the enlargement of the right superior ophthalmic vein (orange arrow).

The patient underwent a new head CT, which was normal. Orbital CT documented slight thickening of the right eyeball upper eyelid soft tissues, slight swelling of the medial and lateral rectus muscles, an increase in the caliber of the superior ophthalmic vein compared to its contralateral, and discrete right eyeball proptosis (Figures 1A, 1B). There was no fever or elevation of inflammatory parameters or other abnormalities in blood tests. The patient was referred to internal medicine and hospitalized for post-septal cellulitis, medicated with intravenous ceftriaxone and vancomycin, and topical treatment, including dorzolamide and timolol eye drops.

**Figure 2:**
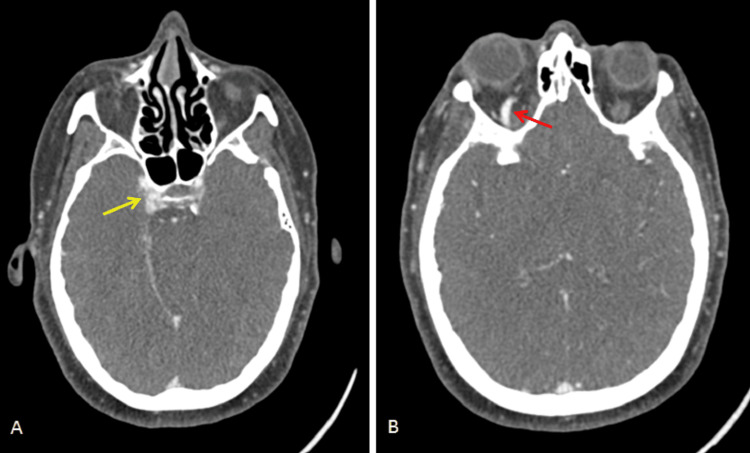
Head angio CT images of the patient. (A) axial view showing the enlargement of the right cavernous sinus due to its arterialization (yellow arrow) and (B) axial view showing the engorgement of the right superior ophthalmic vein due to its arterialization (red arrow).

**Figure 3:**
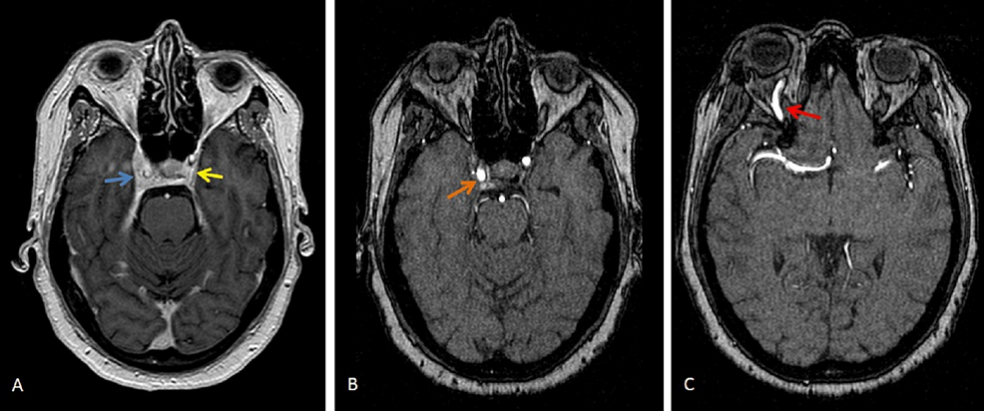
Brain angio MRI images of the patient. (A) Axial view showing the enlargement of the right cavernous sinus (blue arrow) compared to the opposite side (yellow arrow), (B) axial view showing the arterialization of the right cavernous sinus (orange arrow), and (C) axial view showing the arterialization of the right superior ophthalmic vein (red arrow).

On the second day of hospitalization, head CT images were revised by neuroradiology, and an asymmetry of the cavernous sinuses (right>left) was observed, leading to the suspicion of a CCF. So, an angio CT scan was performed for clarification. At this point, due to the exuberant chemosis, it was necessary to start systemic and topical corticosteroid therapy. The angio CT was performed on the third day of hospitalization and revealed an asymmetry of the cavernous sinuses (with the right one being thicker), dilatation of the right superior ophthalmic vein, diffuse thickening of the extraocular muscles, and slight densification of the intra and extraconal fat of the right orbit compared to the opposite side (Figures 2A, 2B). These alterations suggested a CCF. The antibiotics were discontinued. The case was discussed with the interventional neuroradiology team who suggested performing brain magnetic resonance imaging (MRI), whose findings were in favor of CCF diagnosis (Figures 3A-3C).

**Figure 4:**
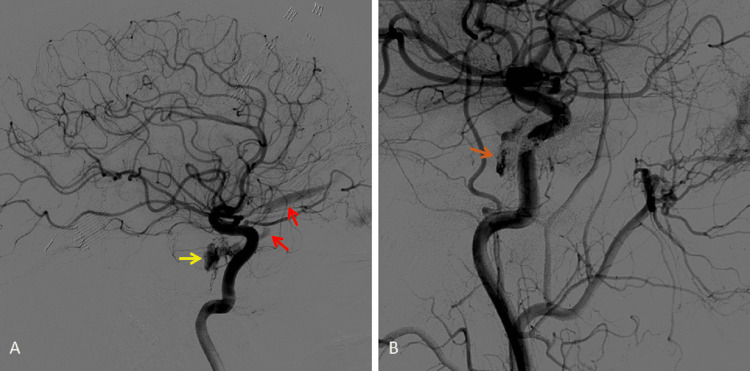
Diagnostic and therapeutic angiography images of the patient. (A) Pre-embolization showing dilatation of the cavernous sinus (yellow arrow) and the superior ophthalmic vein (red arrows) in the arterial phase and (B) after embolization with metallic coils, showing the much less filled cavernous sinus (orange arrow).

None of the medical measures taken so far led to significant improvement in the patient’s symptoms. Diagnostic angiography was performed and confirmed a right cavernous sinus dural fistula supplied by meningeal branches of the internal carotid artery, with drainage into the right superior ophthalmic vein. Embolization with coils of the right cavernous sinus was successfully performed at the same time (Figures 4A, 4B). In the immediate post-procedure period, the patient reported a significant improvement in diplopia and, although not completely resolved, there was clearly less limitation of eye movements.

The patient was discharged two days after the procedure, with no need for any additional systemic or topical treatment. In the ophthalmologic evaluation two weeks later, the patient no longer had diplopia and she had completely recovered the eye movements. There was no chemosis, only slight conjunctival hyperemia, and eyelid edema. Visual acuity had returned to the patient's baseline (20/25). The IOP had dropped to normal values (17 mmHg on both eyes), and there was no vascular tortuosity or retinal lesions on fundoscopy.

## Discussion

The CCFs can be post-traumatic or spontaneous, the latter being a minority and most often due to rupture of intracavernous aneurysms of the ICA [1]. The most widely used CCFs classification system is the one proposed by Barrow et al., which is based on blood supply. According to this system, type A CCFs result from direct communication between the ICA and the CS, they are high-flow shunts and the most common after trauma; types B, C, and D, the indirect (or dural) CCFs, result from communication between meningeal branches of the internal and/or external carotid artery and the CS [5]. Usually, spontaneous indirect CCFs are type D (communication between ICA and ECA branches and the CS) [6]. In type B CCFs, as occurred in our patient, there is communication between the meningeal branches of the ICA and the CS [5]. This type of fistula is rare [1,5].

Indirect types of CCF are more common in women over 50 years of age, with a 7:1 female-to-male ratio [2]. Its presentation can be very diverse, depending on the venous drainage pattern and blood flow velocity. In CCFs with anterior drainage, the major concern is the increase in IOP due to venous congestion with potential impairment of visual acuity [4]. As with our patient, in indirect CCFs there is VI cranial nerve palsy in up to 85% of the cases [1]. This has to do with its central location within the CS, adjacent to the intracavernous portion of the ICA, putting it at greater risk of damage compared to the other nerves that are located in the deep layer of the lateral wall of the CS [6].

The progressive worsening in our patient is consistent with the literature that describes an abrupt presentation in direct fistulas, in contrast to a more indolent clinical presentation in indirect fistulas, which makes them harder to diagnose. Indirect CCFs can be caused by hypertension, fibromuscular dysplasia, type IV Ehlers-Danlos syndrome, or ICA dissection [6]. When our patient went to the ED for the first time, she complained of a headache and had high BP. We suspect that hypertension may have been the genesis of this fistula, although we cannot say it with absolute certainty. In 2019, Law and Docherty described the first case of indirect CCF after an episode of hypertensive emergency. The presentation and evolution of the case were similar to what we describe in this article [7]. Diabetes mellitus has also been described as an underlying etiology of CCFs [2].

The differential diagnosis of CCFs includes cerebral aneurysms, vascular malformations of the eye, post-septal cellulitis, orbit inflammation, exophthalmos due to thyroid disease, retrobulbar hemorrhage, cavernous sinus thrombosis, vasculitis, and lacrimal gland tumor [4]. In the case of our patient, the differential diagnosis with post-septal cellulitis is particularly important. A diabetic patient presented with symptoms that pointed to conjunctivitis. It is well known that infectious conditions in diabetic patients not infrequently evolve unfavorably. Initially, the clinical picture was interpreted as orbital cellulitis that progressed from conjunctivitis. The absence of fever or elevation of inflammatory markers, as well as the absence ofTyndall effect, did not favor the diagnosis, which made us question this hypothesis from the beginning.

The imaging studies for CCFs include CT, angio CT, MRI, and angio MRI. They usually show an asymmetric enlargement of the CS, ophthalmic vein, and extraocular muscles compared to the opposite side [3]. The superior ophthalmic vein is enlarged in 75-100% of the cases - as was the case with our patient - and may be the only imaging finding [1]. The gold standard diagnostic examination is digital subtraction angiography (DSA), as it identifies the location of the fistula, determines its size and classification, and allows endovascular treatment at the same time [3]. Elevated venous pressures and high IOP in patients with CCFs can compromise retinal perfusion, leading to loss of visual acuity, and causing secondary glaucoma with optic nerve damage. Thus, timely diagnosis of CCFs is of utmost importance, so that appropriate treatment can be instituted. All clinicians must be suspicious of the diagnosis, especially when the patient does not respond to conventional topical treatment for conjunctivitis-like symptoms [3].

The treatment decision depends on the severity of the condition [4]. A high number of indirect CCFs resolve spontaneously due to local thrombosis of the superior ophthalmic vein, which spreads posteriorly [2,6]. Thus, in some of these patients with low-flow shunts, the initial medical approach is conservative, using drugs to relieve ocular symptoms and manual intermittent ipsilateral carotid compression. In cases like the one we describe in this article, in which there are secondary ocular complications from CCFs, carotid compression is not recommended, and the treatment should be endovascular or surgical [2,7]. Relief of eye symptoms can help, but, as happened with our patient, definitive treatment is based on reducing venous pressure [3]. In patients with progressive loss of visual acuity, severe proptosis, cranial nerve palsy, IOP>25 mmHg, and engorgement of cortical veins on angiography, endovascular treatment is usually required. The prognosis after endovascular therapeutic approach is excellent [4]. Modern endovascular techniques are highly effective in the treatment of CCFs (90-100% cure), with a low rate of complications and virtually no mortality. However, even with endovascular treatment, the risk of complications is higher and the success rate is lower in indirect CCFs (versus direct CCFs) [6].

## Conclusions

Indirect CCF, although rare, is an important diagnosis to be suspected in patients with a presumptive diagnosis of bacterial conjunctivitis and/or post-septal cellulitis that does not improve with antibiotic treatment. It is the clinical suspicion that leads to a timely diagnosis and treatment, avoiding serious eye damage. This case also demonstrates the importance of a multidisciplinary approach.
